# AIHEMAF–P: An Innovative Healthcare Model for Atrial Fibrillation Patients

**DOI:** 10.3390/pharmacy12060187

**Published:** 2024-12-15

**Authors:** Raffaele La Regina, Pasquale Innelli, Fulvio Glisenti, Gianbattista Bollani, Eugenio Leopardi, Gian Franco Gensini, Savina Nodari, Giuseppe La Regina, Micaela La Regina, Francesco Gabbrielli

**Affiliations:** 1 Farmacia La Regina, 84030 San Rufo, Italy; 2 Cardiovascular Department, Intensive Care Cardiology Unit, San Carlo Hospital, 85100 Potenza, Italy; 3 Research Department, Health Telematic Network, 25124 Brescia, Italy; 4 UTIFAR—Unione Tecnica Italiana Farmacisti, 20124 Milano, Italy; 5 Technical-Scientific Committee and Faculty of Wellness and Life Science, Pegaso International, 1520 Valletta, Malta; 6 Cardiology Section, Department of Medical and Surgical Specialties, Radiological Sciences and Public Health, University of Brescia Medical School, 25123 Brescia, Italy; 7 Laboratory Affiliated to Istituto Pasteur Italia—Cenci Bolognetti Foundation, Department of Drug Chemistry and Technologies, Sapienza University of Rome, 00185 Rome, Italy; 8 Clinical Governance and Risk Management Unit, Azienda Sociosanitaria Ligure 5, 19121 La Spezia, Italy; 9 R&D for Clinical Activity in Telemedicine, Italian National Health Agency—AGENAS, 00187 Rome, Italy

**Keywords:** community pharmacy, atrial fibrillation, telemedicine, POCT, pharmacy services, clinical pathway, case management, interprofessional care, interprofessional cooperation, health system transformation

## Abstract

Atrial fibrillation (AF) is one of the most common cardiac arrhythmias of clinical relevance and a major cause of cardiovascular morbidity and mortality. Following a diagnosis of AF, patients are directed towards therapy with anticoagulant drugs to reduce the thromboembolic risk and antiarrhythmics to control their cardiac rhythm, with periodic follow-up checks. Despite the great ease of handling these drugs, we soon realized the need for follow-up models that would allow the appropriateness and safety of these pharmacological treatments to be monitored over time. This pilot study was conducted at a rural pharmacy. The study comprised 47 patients (average age 71.22 years) with nonvalvular atrial fibrillation (68% being paroxysmal) on NOACs. Twenty percent of the enrolled subjects lived alone and fifty-four percent of the participants stated that they were not independent in managing their treatment. The primary aim was to describe the implementation and the outcomes of an innovative smart clinic model in which a local trained pharmacist is a case manager, and the patient carries out the required checks via telemedicine and point-of-care testing systems (POCT) under the service pharmacy regime; the results of the checks could be shared in real time with the attending general practitioner and the relevant specialist. The secondary aims of this study were to evaluate adherence to the planned controls, the prescriptive appropriateness of the dosages and drugs and adherence to the prescribed therapy, the occurrence of pharmacological problems linked to drug type interactions, the occurrence of hemorrhagic and/or thromboembolic complications, the acceptance by the general practitioners and/or the specialists of the reports made by the pharmacist on the subsequent actions undertaken, the economic and social impact of this model on the National Health Service and on the patient, and the impact on the quality perceived by the patients involved in this innovative monitoring process. Compliance with the planned checks was 93%. The dosage of the anticoagulant drug during enrollment was found to be inappropriate, without apparent clinical reasons, in 11% of the sample. Adherence to the anticoagulant therapy was found to be 98%. In total, 214 drug–drug interactions of varying clinical relevance were detected. No embolic events were detected; however, 13% of the sample reported a major hemorrhagic event, which came to light thanks to the close monitoring of hemoglobinemia. A total of 109 reports were made to the patients’ referring doctors in relation to the summarized anomalies, and 84% were accepted by the referring clinicians. Therefore, community pharmacists and pharmacy services represent ideal actors and contexts that, when integrated into the care network, can really favor individual care plan adherence and achieve daily morbidity reductions and cost savings through proper disease control and the early diagnosis of complications.

## 1. Introduction

Atrial fibrillation (AF) is one of the most common cardiac arrhythmias of clinical relevance and one of the main causes of cardiovascular morbidity and mortality. It represents one of the most important risk factors for ischemic stroke and is characterized in these cases by a more severe prognosis in terms of survival and residual disability.

Italy is one of the countries with oldest people in the world, with a percentage of over-65s that exceeds 21%; therefore, age-related disorders, such as AF, are of great importance for the National Health Service, and their impact will increase with population aging [1].

Recently, a study, born with the aim of estimating the prevalence of AF in the Italian population and of generating projections for Italy and the European Union, predicted the following:

- The number of elderly Italian people affected by AF will rise from the current level of approximately 1.1 million to 1.9 million in 2060;
- The number of elderly Europeans affected by AF will rise from the current level of approximately 7.6 million to 14.4 million in 2060.

This study therefore indicates an increase in the burden of AF in the coming decades, especially among the elderly, who have a higher risk of stroke and complications related to AF.

Following a diagnosis of AF, a patient is usually treated with anticoagulant drugs, to reduce the thromboembolic risk, and antiarrhythmics, to control their cardiac rhythm, with periodic follow-up checks [2,3].

Usually, for this therapeutic indication, new-generation oral anticoagulant drugs (NOAC), like edoxaban, rivaroxaban, apixaban, and dabigatran, are used.

When these drugs were introduced as an alternative to the now well-known warfarin, their diffusion was very fast due to the lack of need for frequent blood coagulation monitoring and fewer drug–food and drug–drug interactions.

However, it soon became evident that there was a need to develop new monitoring models that, even with a lower frequency than those used for warfarin, would allow the practiced therapy to be evaluated over time to confirm the appropriateness and safety of the treatment. In this regard, in countries where the use of electronic health records is already widespread, studies were immediately conducted to determine which elements should be monitored and re-evaluated in the follow-up phase through the implementation of dedicated dashboards and ad hoc protocols in order to expand the services provided by anticoagulation clinics [4,5].

In the follow-up plans of these patients, much attention has always been paid to renal function and its related hemorrhagic consequences. In fact, people with renal failure are at a greater risk of hemorrhagic and/or thromboembolic events, and this risk increases over time. For this reason, a continuous reassessment of renal function is necessary to ensure that the patient is consistently administered the appropriate dose of anticoagulants [6,7].

The activities performed by community pharmacists are particularly useful for this reassessment, as highlighted by the abundant evidence in the literature. In fact, the intervention of community pharmacists, owing to their continuous contact with patients and specific training, can ensure the appropriateness, safety, and effectiveness of the practiced pharmacological therapy [8,9,10,11,12,13,14].

The involvement of community pharmacists, as described by the evidence in the literature, is possible when care models providing the complete and standardized integration of the different actors in the care chain are created. Scenarios of this type are now consolidated in various countries around the world, but in Italy, they are still struggling to come to life [15,16,17,18,19,20,21,22,23].

In Italy, access to anticoagulant therapy is possible only after the drafting of an electronic treatment plan, which, in the past, could only be issued by a specialist. After the introduction of the prescriptive appropriateness note 97 by the Italian Medicines Agency (AIFA), general practitioners were also given the opportunity to take charge of patients being treated with NOAC in order to simplify the follow-up activities related to anticoagulants.

Despite all of these improvements to follow-up care, due to the way that the National Health Service is organized today, the lack of reconciliation between diagnostic–therapeutic activities, the pace of life, and the social context of patients create lose–lose scenarios.

Therefore, it becomes necessary to design modern care models based on the use of digital devices and telecommunication systems and thus minimize the time needed to deliver the relevant services. This is the most realistic way to achieve territorial integration of all the care settings and all their available healthcare professionals. This is to enable them to carry out the activities envisaged in their care plans in the simplest and most immediate way with the treatments best suited to their state of health, responding effectively and efficiently to emerging needs.

In the care chain, one of the professionals that is still seldom considered in Italy today is the community pharmacist, who, despite their position within the National Health Service, represents an underutilized partner, especially in telecare for patients with chronic illnesses.

Nevertheless, starting from 2009, through a series of legislative acts, a profound transformation of the professional profile of the community pharmacist was initiated based on the desire to allow them to acquire new skills related both to so-called ‘cognitive services’ (monitoring of therapy adherence, pharmacological recognition, and reconciliation, health information, etc.) and to instrumental diagnostic support (provision of telemedicine services, point-of-care testing (POCT), etc.).

In the light of these changes, both in patient care needs and in the professional profile of the community pharmacist, the pharmacist could also be entrusted with the role of case manager (a professional figure already existing in other countries, who manages one or more cases entrusted to him or her according to a pre-established protocol, such as the diagnostic–therapeutic care pathway, in a defined space–time context).

By guaranteeing safety, the pharmacist’s activity as case manager could be tried in Italy for patients suffering from AF and being treated with new-generation oral anticoagulants. In this scenario, the community pharmacist would usefully support local health services without replacing any of the other health professionals already present in the care chain and could facilitate both de-hospitalization and the territorial management of patients with chronic diseases.

## 2. Materials and Methods

### 2.1. Design

The study covered by this work was observational, prospective, monocentric, and not for profit. Due to the concomitant COVID-19 epidemiological emergency, enrollment took place in a staggered manner over time, and therefore, the study took place from November 2021 to March 2023. Each patient, however, was followed up for a period of 12 consecutive months.

The primary endpoint of this study was to describe the implementation and outcomes of an innovative smart clinic model that would be useful for the clinical and pharmacological monitoring of patients suffering from nonvalvular AF who were being treated with new-generation oral anticoagulant drugs. In this model, an adequately trained community pharmacist acted as a case manager and the patient had the opportunity to undergo the follow-up activities envisaged by their treatment plan through the use of telemedicine and POCT systems. The outcomes of the activities were shared in real time with the general practitioner and the reference specialist to define any necessary future actions.

The secondary endpoints of the study were as follows:

- An assessment of compliance with the planned controls;
- An evaluation of the prescriptive appropriateness of the dosages and molecules used;
- An assessment of adherence to the prescribed therapy;
- An evaluation of the occurrence of pharmacological problems linked to drug–drug interactions;
- An evaluation of the occurrence of hemorrhagic and/or thromboembolic complications;
- An evaluation of acceptance by the general practitioner and/or the specialist of the reports made by the pharmacist and of the subsequent actions undertaken;
- An evaluation of the economic and social impact of this model on the National Health Service and on the patient;
- An evaluation of the impact on the quality perceived by patients in carrying out this innovative care model.

### 2.2. Sample and Setting

To conduct this study, a sample size of 50 patients was defined.

All the patients provided informed consent before participating in the study. The inclusion criteria were (1) age > 18 years, (2) a diagnosis of nonvalvular AF, (3) treatment with new-generation oral anticoagulant drugs, (4) the ability to consent to the study, and (5) an up-to-date anti-COVID-19 vaccination.

This study was conducted in a rural community pharmacy affiliated with the National Health Service and involved 13 general practitioners and 10 cardiology specialists.

### 2.3. Intervention

The study design included the following phases:

- Phase 1: Patient enrollment (November 2021–February 2022): in the first phase, which lasted four months due to the concomitant health emergency, the patients were enrolled.
- Phase 2: Review, database validation, and definition of individual assistance plans (November 2021–February 2022): in this phase, the patient database was validated, and individual care plans (ICPs) were defined by the general practitioners.
- Phase 3: Case management and follow-up (November 2021–February 2023): during this phase, the case manager pharmacist followed up with the patients and carried out the checks required by the current guidelines and by the ICP drawn up by the treating doctor in case the follow-up path needed to be personalized due to the particular and specific clinical conditions of the patient (e.g., impaired renal function, lack of rhythm control, etc.). All 12-lead electrocardiogram (ECG), Holter ECGs, and blood tests were carried out directly in the pharmacy using telemedicine and POCT systems (ECG and Holter Mortara, POCT Allegro Nova Biomedical).

After each follow-up meeting, a report, including all the measurements that were carried out, was sent to the attending physician and/or the reference specialist, who, based on the clinical history of the patient in question, defined the actions to be taken.

For the services provided by the ICP but not available at the pharmacy, the pharmacist proceeded by requesting the necessary prescription from the general practitioner, booking the test, and providing a telephone reminder service to remind the patient of when to carry out the test.

### 2.4. Data Collection

For each patient, the clinical information, together with organizational and management information, was collected in a case report form.

In order to collect homogeneous data, the structure generally included the following macro-areas:

- General information about the patient (ID, sex, age, etc.);
- Information relating to the family setting (spouse, children, caregivers, etc.);
- Immediate and remote clinical history;
- Pharmacological therapy;
- Vital signs and initial blood tests;
- Tests and procedures required for follow-up;
- Results and reports of checks carried out during follow-up;
- Perceived quality questionnaire.

Furthermore, during each meeting, a form dedicated to collecting the following information was filled out:

- State of health since the last pharmacological refill;
- Adherence to anticoagulant therapy;
- Bleeding and risk factors for bleeding;
- Creatinine clearance/kidney function;
- Drug interactions;
- Final exam/evaluation.

### 2.5. Data Analysis

Adherence to the checks was assessed by referring to the meetings scheduled by the ICP, and adherence to the therapy was assessed monthly by manually counting the residual dosage units.

The statistical analysis of the collected data was developed through a descriptive analysis.

The economic evaluation was carried out by evaluating the savings that could be generated through the implementation of this innovative model, making reference to the regional tariff.

The assessment of perceived quality was carried out using a short anonymous questionnaire administered at the end of the study to all the enrolled subjects.

All the categorical variables measured in this study are expressed as an absolute number and percentage. Continuous variables are expressed as a mean and standard deviation or a median and interquartile range, depending on whether the data were normally distributed.

Differences were analyzed using chi-square tests, unpaired *t*-tests, or Mann–Whitney U tests, depending on the nature of the variable.

### 2.6. Ethical Considerations

This study was conducted in accordance with the Declaration of Helsinki, and the protocol was approved by the Campania Sud ethics committee (authorization no. 125, 8 September 2021) and registered on clinicaltrials.gov (NCT05086991).

## 3. Results

### 3.1. Patient Characteristics

A total of 50 patients were enrolled (46% women; mean age, 71.22 ± 10.71 years). Of the patients enrolled, three withdrew their consent before the start of the activities for fear of COVID-19 infections, as the activities took place during the emergency, and one patient withdrew consent halfway through the research process.

Descriptive data of the study population are summarized in Table 1.

In total, 20% of the enrolled subjects declared that they lived alone and 54% of the participants declared that they were not independent in managing their treatment path.

### 3.2. Primary Endpoint

The primary objective of this study was to test the designed care model based on the smart clinic idea, as it would be useful for the clinical–pharmacological monitoring of patients suffering from nonvalvular AF who were being treated with new-generation oral anticoagulant drugs; for this study, an adequately trained pharmacist took on the role of case manager. The necessary diagnostic tests were carried out owing to the use of telemedicine systems (ECG and Holter ECG, reported by the remote cardiology specialists) and POCT (blood tests).

After enrollment, each patient underwent the following preliminary investigations:

- 12-lead ECG;
- Evaluation of drug interactions using the Intercheckweb platform of the Mario Negri Institute;
- 24 h Holter ECG;
- Blood pressure measurements;
- Blood chemistry tests (creatinine, hemoglobin, and hematocrit).

Subsequently, the following information was collected using a checklist created by Thrombosis Canada:

- State of health since last refill of the anticoagulant drug, in order to ascertain whether the patient had been subjected to unexpected and/or planned procedures/visits/investigations;
- Adherence to the anticoagulant therapy, in order to obtain information on adherence to therapy and any improper use of the drug;
- Bleeding and risk factors for bleeding, in order to ascertain whether bleeding events had occurred and to monitor the trend of the hemoglobin values and the appearance of risk factors for bleeding (drinking, falls, uncontrolled blood pressure, etc.);
- Creatinine clearance and renal function, in order to obtain information on the appearance of alterations in renal function and/or predisposing factors (e.g., vomiting, diarrhea, etc.) and on the trend in creatinine and GFR values;
- Pharmacological interactions, in order to evaluate the presence of critical issues related to the interaction between the NOAC and other drugs taken;
- Examination/evaluation, in order to collect information, through measurements, on the patient’s blood pressure and the occurrence of any symptomatic episodes of hypotension.

Based on the collected information, the pharmacist highlighted the data of greatest significance in relation to the patient’s needs and sent them all in electronic format to the patient’s general practitioner and cardiology specialist in order to define the ICP, which contained indications on the activities (ECG, blood tests, etc.) that the patient had to undergo in follow-up meetings and their frequency.

Follow-up meetings took place on a monthly basis when the anticoagulant drug was collected from the pharmacy.

At each meeting, the pharmacist case manager again filled out the check list described previously and carried out the investigations requested by the patient’s referring doctors and/or deemed necessary based on the reports of alterations in the patient’s state of health (e.g., assessing creatinine levels in potentially dehydrated patients, hemoglobinemia).

After each meeting, the pharmacist case manager sent the collected data to the patient’s referring doctors, who then indicated any actions to be taken based on the outcome of the meeting.

### 3.3. Secondary Endpoints

Compliance with the planned checks was 93%.

The dosage of the anticoagulant drug, during enrollment, was found to be inappropriate (dosage not related to renal function), without apparent clinical reasons, in 11% of the sample.

Adherence to anticoagulant therapy was found to be 98%.

A total of 214 drug–drug interactions of varying clinical relevance were detected, as summarized in Table 2.

No embolic events were detected; however, 13% of the sample reported major hemorrhagic events, which came to light thanks to the close monitoring of hemoglobinemia and were resolved thanks to rapid intervention.

A total of 109 reports were made to the patients’ referring doctors in relation to the anomalies summarized in Table 3, and 84% were accepted by the referring clinicians.

Due to the innovative nature of the model, precise and timely estimates of its costs were not possible. In fact, at present, in Italy, community pharmacists are not officially entrusted with chronic patient care; for this reason, it is impossible to evaluate the cost of this activity for the public National Health Service.

With regard to the diagnostic tests foreseen by the individual care plans, the costs of the tests carried out in the pharmacy are very much comparable to those already charged by the National Health Service through the provision of the same tests by public structures and affiliated private clinics.

The multiple-choice questionnaire, which was aimed at measuring perceived quality, is summarized in Table 4.

## 4. Discussion

Currently, a patient suffering from AF cannot easily adhere to their treatment program due to the current organization of the Italian National Health Service.

Various critical issues detectable in the structural and organizational aspects of the current treatment paths undermine adherence to follow-up appointments.

In fact, at present, the patient is forced to interact with many, perhaps too many, actors in the care chain and to go to just as many healthcare facilities.

Consequently, failure to adhere to checks generates a delay in identifying situations that would require corrective interventions by doctors, and it also exposes the patient to risks linked to drug therapy that could be ineffective or even dangerous.

This latter event, which is not uncommon, may be the result of a worsening of the patient’s physiological parameters, which require an adjustment of their therapy in terms of dosage, posology, and/or the outcome of any drug–drug interactions; this is a common event in poly-treated patients.

Based on these reasons, we decided to design and test this care model in order to provide patients with a smart clinic model serving as a follow-up tool that would allow them to monitor their state of health and the progress of their drug therapies in the easiest way possible, taking advantage of the widespread presence of pharmacies in the area, the most innovative medical technologies, and the network of health professionals.

This vision has proven to be successful, as demonstrated by the adherence data, as it has allowed not only patients of working age but also elderly people to adhere to the controls, allowing them to reconcile personal care with work commitments and allowing them to have greater autonomy by not being forced to face long and numerous journeys and having to resort to the help of third parties, respectively.

As can be easily seen from the results shown previously, continuous contact with a reference figure represents the key point of this model.

In fact, patients currently lack a constant reference in their treatment path that guides them, listens to them, and directs them correctly towards the health services that they need.

First of all, continuous contact with a reference figure allows the continuous collection of information related to the social status, life rhythms, and/or circumstances that may have direct and/or indirect consequences on adherence to the path and/or therapy.

A further advantage of the model under study is the possibility of having, owing to telemedicine and POCT tools, continuous information on the patient’s health status.

This also represents an undoubted advantage for the patient’s medical personnel (general practitioners and specialists), as it allows them to continuously receive information on their patients, avoid improper access to clinics, and intervene promptly in the event of situations requiring corrective actions, which, as seen in the experiment, can often be implemented without recourse to outpatient and/or emergency services.

As an example, consider how the bleeding events that occurred during this study were managed. The continuous monitoring of hemoglobin made it possible to highlight its negative trend and to begin the necessary investigations to ascertain its origin.

A similar argument can be made for patients who, owing to the continuous monitoring of their renal function, had their anticoagulant or antiarrhythmic dosage reduced after ECG Holter monitoring.

Another interesting aspect that emerged during the trial is linked to the pharmacist’s continuous evaluation of drug–drug interactions. As the results clearly show, such events are not uncommon and can often create critical situations, especially when the patient follows a complex therapy that also includes drugs with a narrow therapeutic index for rhythm control.

Regarding the economic aspects, it was not possible to quantify the cost associated with case management, as this activity is not currently included in the tariff nomenclature. However, calculating it in a subsequent study could be of interest. As for the cost of diagnostic services, the expense of a test performed in a pharmacy is quite comparable to that of one performed in a hospital or outpatient facility.

If, however, the costs of implementing a model like this on a large scale are considered, we will certainly witness a dramatic and exponential increase in spending linked to greater accessibility to services by patients. However, we are confident that, in the medium to long term, the return on investments generated by the savings from the reduced improper use of outpatient and/or emergency services, the early diagnosis of complications, and the timely correction of physiological parameters that deviate from the optimal levels defined by clinicians will be notable.

Regarding the quality of the model under study, as perceived by the patient, the pharmacist appears to be recognized as a reference figure and the diagnostic services as welcome, effective, and easily accessible.

The monometric nature of the study and the small number of patients certainly represent the limitations of this study. However, the biggest limitation is the absence of a control group. The absence of a control group

- Makes it impossible to determine whether the results obtained are attributable to the innovative model or to other factors, such as, for example, patient motivation or external healthcare improvements;
- Makes it difficult to identify any confounding variables, such as, for example, patient demographics, disease severity, or baseline adherence rates;
- Generates interpretative difficulties in evaluating the ability of this model to overcome traditional treatment paths.

Based on these observations, however, it is possible to hypothesize the design and start of a new study with a greater number of patients, a greater number of participating pharmacies, and the presence of a control group.

## 5. Conclusions

As noticed in the examined literature, this is the first Italian study to investigate the possible role of community pharmacists in the care of patients suffering from AF who are being treated with new-generation anticoagulants.

The results show that a community pharmacist can be a new and strategic partner of the National Health Service in the care of patients with chronic illnesses.

The adoption of models, such as the one examined above, for the various chronic pathologies with which the Italian population appears to be affected will certainly favor patients’ adherence to treatment paths and will facilitate a reduction in costs as a consequence of the early diagnosis of complications, as well as a reduction in the improper use of health services.

However, to make all of this a reality, changes need to be implemented in the following areas:

- Education: universities need to build courses on these new clinical/organizational skills that society will demand of community pharmacists and provide advanced training courses for existing professionals.
- National Health Service: a profound transformation of the treatment pathways is necessary to better ensure patient adherence.
- Technological infrastructures: technologies that allow the patient to always be in contact with the relevant clinicians become necessary as they make it possible to collect clinical data in every care setting and to make predictions on the health status of patients using artificial intelligence systems.
- Care supply chain: the community pharmacist should be recognized as a strategic partner by the other actors in the supply chain, and their role should be defined unequivocally and precisely in order to avoid counterproductive overlaps, as should the relationships between different professionals in order to best define freedom of action and responsibility.

## Figures and Tables

**Table 1:** Patient characteristics at baseline (n = 50).

Characteristics	n (%)
Demographic data	
Sex, female	23 (46%)
Age, mean (SD)	71.22 (10.71)
Health status	
Comorbidities, median (SD) (HIV infection, dementia, neoplasm, liver disease, kidney disease, peripheral vascular disease, respiratory disease, cardiovascular disease, diseases of the nervous system, non-neoplastic hematological diseases, rheumatic diseases, gastro-entero-pancreatic diseases, or other diseases)	2.0 (1.4)
Type of atrial fibrillation	
Paroxysmal atrial fibrillation	34 (68)
Persistent atrial fibrillation	0 (0)
Long-lasting persistent atrial fibrillation	3 (6)
Permanent atrial fibrillation	13 (26)

**Table 2:** Detected drug–drug interactions.

Clinical Relevance	N (%)
A. Low	4 (1.87)
B. Moderate	137 (64.02)
C. Severe	52 (24.30)
D. Very Severe	21 (9.81)

**Table 3:** Clinical events detected.

Reported Anomaly	N (%)
ECG	24 (22.02)
Holter ECG	24 (22.02)
Blood pressure > 160 mmHg	30 (27.52)
GFR reduction	6 (5.50)
Hemoglobin reduction	10 (9.17)
High anticoagulant dose	6 (5.50)
Low anticoagulant dose	5 (4.59)
Therapy adherence problem	4 (3.67)

**Table 4:** Results of the perceived quality assessment.

Questions	Answers		
	%	N	Choices
Are you aware of the investigations that must be carried out every year to control this disease and its chronic complications?	100	47	Yes
	0	0	No
Who informed you about the diagnostic therapeutic path to be followed?	54	26	General practitioner
	96	45	Community pharmacist
	64	30	Cardiologist
In the last two years, have you carried out all the required checks?	0	0	Yes
	100	47	No
Based on your experience in this study, do you believe that a pharmacist could be the most suitable figure to follow up with you in your treatment path and to help control your disease?	96	45	Yes
	4	2	No
What did you appreciate most about the activities carried out by the pharmacist during this study?	85	40	Point-of-care testing
	79	37	Telemedicine services
	47	22	Reminder service
	51	24	Counseling service
	68	32	Constant availability
	57	27	Clarity of the provided information
	83	39	Ease of access to services

## Data Availability

The data presented in this study are available on request from the corresponding author due to privacy and legal reasons.
